# Self-rated health, interviewer-rated health, and objective health, their changes and trajectories over time, and the risk of mortality in Chinese adults

**DOI:** 10.3389/fpubh.2023.1137527

**Published:** 2023-06-20

**Authors:** Shiyi Shan, Jin Cao, Ke Tang, Siqing Cheng, Ziyang Ren, Shuting Li, Weidi Sun, Leying Hou, Qian Yi, Dingwan Chen, Peige Song

**Affiliations:** ^1^School of Public Health and Women’s Hospital, Zhejiang University School of Medicine, Zhejiang University, Hangzhou, Zhejiang, China; ^2^School of Public Health, Zhejiang University School of Medicine, Zhejiang University, Hangzhou, Zhejiang, China; ^3^Department of Orthopedic Surgery, The Forth Affiliated Hospital, International Institutes of Medicine, Zhejiang University School of Medicine, Yiwu, Zhejiang, China; ^4^School of Public Health, Hangzhou Medical College, Hangzhou, Zhejiang, China

**Keywords:** self-rated health, interviewer-rated health, the multimorbidity-weighted index, Chinese older adults, mortality

## Abstract

**Background:**

Self-rated health (SRH), interviewer-rated health (IRH), and objective health reflect the overall health status from different aspects. This study aimed to investigate the associations of SRH, IRH, and objective health with mortality among Chinese older adults.

**Methods:**

This study used data from the 2008 (baseline), 2011, 2014 and 2018 waves of the Chinese Longitudinal Healthy Longevity Survey. SRH and IRH were evaluated by questionnaire. Objective health was evaluated by the Chinese multimorbidity-weighted index (CMWI), which incorporated 14 diagnosed chronic diseases. SRH, IRH, and CMWI were assessed as: (1) baseline levels; (2) longitudinal changes by subtracting the values obtained in 2008 from the corresponding values in 2014; (3) trajectories by Group-Based Trajectory Modeling, respectively. The Cox proportional hazards model was used to explore the associations of baseline SRH, IRH, and CMWI, their changes, and trajectories with mortality.

**Results:**

A total of 13,800 participants were included at baseline (2008). The baseline SRH ([hazard ratio] 0.93, [95% confidence interval] 0.91–0.96), IRH (0.84, 0.81–0.87), and CMWI (0.99, 0.98–1.00) in 2008 were significantly associated with 10-year mortality (2008 to 2018). Among 3,610 participants, the changes of SRH (0.93, 0.87–0.98), IRH (0.77, 0.71–0.83), and CMWI (0.97, 0.95–0.99) from 2008 to 2014 were significantly associated with 4-year mortality (2014–2018). The trajectories were divided into “high SRH/IRH/CMWI” and “low and declining SRH/IRH/CMWI.” Compared with “low and declining SRH/IRH/CMWI,” “high SRH” (0.58, 0.48–0.70), “high IRH” (0.66, 0.55–0.80), and “high CMWI” (0.74, 0.61–0.89) from 2008 to 2014 were significantly associated with 4-year mortality (2014–2018).

**Conclusion:**

Baseline SRH, IRH, and CMWI, their changes and trajectories are all associated with mortality in Chinese older adults. It is possibly necessary to promote the use of cost-effective indicators in primary medical institutions to improve the health management of the older adults.

## Introduction

With the declining fertility rate and the development of modern medical care, the aging process in China accelerates (1). According to the Seventh National Population Census of the People’s Republic of China, in 2020, the people aged 60 and above, 65 and above accounted for 18.70 and 13.50% of the total Chinese population, respectively (2). It is projected that by 2050, the proportion of people aged 65 and above will increase to 26.9% of the total Chinese population (3). Meanwhile, as the prevalence of chronic diseases among the older adults continues to grow, the associated burden on public health and socioeconomic development becomes more severe (4). Hence, cost-effective health indicators for the older adults are expected to help public health institutions identify high-risk groups and allocate resources effectively to improve health management in an aging society.

In recent years, simplified indicators of health status have gained attention from researchers. One such indicator is Self-Rated Health (SRH), which measures respondents’ own evaluation of their health status, and encompasses physical health, mental health, and social participation simultaneously (5, 6). Previous studies have confirmed the reliability of SRH in predicting overall health (7), and the World Health Organization (WHO) has recommended SRH as a cost-effective and reliable surrogate indicator of health status (8). However, SRH may exhibit disparities among individuals due to a set of cognitive factors, such as different understanding of health. Interviewer-Rated Health (IRH) addresses this limitation by enabling interviewers to evaluate respondent’s health status during interviews (9). Interviewers often have a more comprehensive and objective perception of health than respondents themselves, and thus IRH can provide supplemental information to SRH, such as respondents’ living environments and lifestyle habits (9). However, these subjective health indicators may lack specificity and pose challenges in ensuring the accuracy of information, particularly when they are used to determine eligibility for further care resources (10). In contrast to subjective health, objective health refers to the health status of respondents characterized by objectively measured biochemical or functional indicators. Recently, the Chinese multimorbidity-weighted index (CMWI) has been proposed as an objective health evaluation index that can quantify the burden of multimorbidity among Chinese older adults through routinely physical examinations at the community health care center (11).

Previous studies have illustrated that poor subjective and objective health were associated with an increased risk of mortality in older adults (12–18). A study involving 373,761 participants from the UK Biobank investigated the associations of different combinations of SRH and objective health status with all-cause mortality, and found that individuals with concordant favorable SRH and health status had the lowest hazard of mortality, while those with concordant unfavorable SRH and health status had the highest hazard of mortality, and those with discordant SRH and health status had intermediate hazards of mortality (19). However, studies also indicated that health status is dynamic and can change over a long follow-up period (20). Some studies have explored the associations of the change and trajectory of SRH with mortality, but the findings have been inconsistent (21–24). For instance, the Lifelines Cohort Study suggested that older adults individuals with a stable poor SRH trajectory often had unfavorable health status (21). While another study with 3,129 participants indicated no significant associations between SRH trajectories and mortality (24). Besides, it remains unclear whether changes and trajectories of IRH and CMWI over time are associated with mortality.

To fill this research gap, we aimed to investigate (1) the association of baseline SRH, IRH, and CMWI with mortality; (2) the association of changes of SRH, IRH, and CMWI (the values of SRH, IRH, and CMWI in 2014 minus the values in 2008) with mortality; (3) the association of trajectories of SRH, IRH, and CMWI (constructed by the values of SRH, IRH, and CMWI in 2008, 2011, and 2014) with mortality.

## Methods

### Study design and participants

The data used in this study came from the 2008, 2011, 2014, and 2018 waves of the Chinese Longitudinal Healthy Longevity Survey (CLHLS), an ongoing and national prospective cohort study of community-dwelling Chinese older adults, which aims to investigate factors related to health and longevity. Details of the CLHLS have been reported elsewhere (25–28). The baseline survey in 2008 consisted of face-to-face questionnaire interviews (including sociodemographic, SRH, IRH, medication history and medical history), physical measurements (including blood pressure, height and weight), and blood sample collection. Standard procedures were followed by well-trained examiners (27).

The CLHLS was approved by The Research Ethics Committees of Peking University (IRB00001052-13074) and Duke University Health System’s Institutional Review Board. Informed consent was obtained from all respondents or their proxy respondents.

### Measures

#### SRH

SRH was defined using the following question, “How do you rate your health at present?” Responses to this question were graded with options of “very good,” “good,” “fair,” “poor,” “very poor,” and “unable to answer.” We combined “poor” and “very poor” considering small frequencies, and recorded “unable to answer” as “missing” in the analysis. The categories of “poor/very poor,” “fair,” “good,” and “very good” were assigned 1–4 points in turn.

#### IRH

IRH was defined using the question, “how does the older adults look?” to rate the respondent’s overall health by interviewers during each wave. The options, including “moderately or severely ill,” “slightly ill,” “relative healthy,” or “healthy,” were given 1–4 points in turn.

#### CMWI

The development process of CMWI has been described in detail elsewhere (11). Briefly, CMWI was developed using physical functioning (PF) as the outcome variable and 14 chronic diseases as predictors, based on data from the China health and retirement longitudinal study. A linear mixed model was used to quantify the weights of each disease in the multimorbidity burden. The effect of each chronic disease on PF was measured with the relevant regression coefficient after adjusting for age, sex and other chronic diseases, and the absolute value of which was denoted as the weight of the disease. For the calculation of CMWI, the following 14 chronic diseases were assigned a score (X_1_ to X_14_) of 1 (yes) or 0 (no) depending on whether the participant had a disease: stroke, memory-related diseases (dementia and Parkinson’s disease), cancer or malignant tumor, asthma, arthritis or rheumatism, emotional, nervous or psychiatric problems, heart disease, chronic lung diseases, hypertension, kidney disease, diabetes or high blood sugar, stomach or other digestive disease, dyslipidemia and liver disease.

Blood pressure was measured two times at a 1-min interval. The average of the two measurements was calculated to the nearest 1 mmHg for analysis. Hypertension was defined as: (1) systolic blood pressure ≥ 140 mmHg and/or diastolic blood pressure ≥ 90 mmHg, and/or (2) self-reported having been diagnosed with hypertension, and/or (3) being treated for hypertension. A 5 mL fasting venous blood sample was collected in heparin anticoagulant vacuum tubes by veteran medical staff. The local Center for Disease Control and Prevention was in charge of routine blood and urine testing. The samples were then transported to Beijing using the specific transport boxes. The central laboratory at Capital Medical University analyzed the samples and tested indicators including four items of blood lipid tests (triglyceride, total cholesterol, high density lipoprotein cholesterol, low density lipoprotein cholesterol) and blood glucose. Diabetes and high blood sugar were defined as: (1) fasting blood glucose >6.1 mmoL/L, and/or (2) self-reported having been diagnosed with diabetes, and/or (3) being treated for diabetes. Dyslipidemia was defined as: (1) triglyceride >1.71 mmoL/L, and/or (2) total cholesterol >5.18 mmoL/L, and/or (3) high-density lipoprotein <1.04 mmoL/L, and/or (4) low-density lipoprotein >3.10 mmol/, and/or (5) self-reported dyslipidemia, and/or (6) being treated for dyslipidemia. Other diseases were defined as self-reported having been diagnosed by doctors and/or being treated for corresponding diseases. The value of CMWI was calculated using the following formula (11):


$$\begin{array}{l} \mathrm{CMWI}=\left(-5.1\right)X_{1}+\left(-4.3\right)X_{2}+\left(-3.4\right)X_{3}+\left(-2.4\right)X_{4}\\ \;+\left(-2.2\right)X_{5}+\left(-2.1\right)X_{6}+\left(-1.7\right)X_{7}+\left(-1.6\right)X_{8}\\ \;+\left(-1.3\right)X_{9}+\left(-1.1\right)X_{10}+\left(-1.0\right)X_{11}+\left(-0.7\right)X_{12}\\ \;+\left(-0.2\right)X_{13}+\left(-0.2\right)X_{14} \end{array}$$

where X_i_ referred to the score of 1 (yes) or 0 (no) depending on whether the participant had the following 14 diseases: stroke, memory-related diseases (dementia and Parkinson’s disease), cancer or malignant tumor, asthma, arthritis or rheumatism, emotional, nervous or psychiatric problems, heart disease, chronic lung diseases, hypertension, kidney disease, diabetes or high blood sugar, stomach or other digestive disease, dyslipidemia, and liver disease.

#### Death

The participant’s survival status and/or date of death was collected during the follow-up (2011, 2014, and 2018). The death of the participants was confirmed and determined by a close family member or a doctor.

#### Covariates

Covariates included socio-demographic characteristics (e.g., age, sex, residence, education, annual income, marital status, physical activity, smoking, and drinking history) and body mass index (BMI). Age was defined as continuous variables. Residence was classified as rural and urban. Education was divided into illiteracy, primary education, and middle school or above. Annual income had three categories: CNY 0–29,999, CNY 30,000–59,999, and > CNY 60,000. Marital status included married or cohabitation, and unmarried or widowed. Physical activity was divided as no and yes. Smoking history was classified as never smoking and ever smoking (including current smoking). Similarly, drinking history was classified as never drinking and ever drinking (including current drinking). BMI (kg/m^2^) was calculated as weight (kg) divided by the square of height (m), which was further analyzed as a continuous variable.

### Statistical analysis

The baseline characteristics were described as the mean with standard deviation (SD) for normally distributed continuous variables, the median with interquartile range (IQR) for nonnormally distributed continuous variables, and numbers with percentage (%) for categorical variables. Wilcoxon rank-sum tests for continuous variables and Chi-square tests for categorical variables were used to evaluate the differences between participants who have died and who survived or lost to follow-up. Three analyses were conducted:

First, a multivariable Cox proportional hazards model with hazard ratios (HRs) and 95% confidence intervals (CIs) with the follow-up time in years as the time scale was used to explore the associations of baseline SRH, IRH, and CMWI (2018) as continuous variables with 10-year mortality (2008–2018). Model 1 was unadjusted; Model 2 was adjusted for age and sex; Model 3 was further adjusted for residence, education, annual income, marital status, physical activity, smoking history, drinking history, and BMI based on model 2. Sensitive analyses were also conducted to investigate the associations of SRH and IRH as categorical variables with 10-year mortality as a robustness check. Since the associations between SRH, IRH, CMWI and mortality may be different between males and females, the sex-stratified multivariable logistic regression was conducted as well (29).

Second, the multivariable Cox proportional hazards model was used to explore the associations between the changes of SRH, IRH, CMWI (2008–2014) and the 4-year mortality (2014–2018). The changes of SRH, IRH, and CMWI were defined as the values in 2014 minus the values in 2008. Improvements of health were those that had a change value larger than zero. All models and adjustments were consistent with those above.

Third, the trajectories of SRH, IRH, and CMWI in 2008, 2011, and 2014 were plotted and grouped using Group-Based Trajectory Modeling (GBTM). GBTM can divide the whole population into individual clusters with similar trajectories according to the trajectory of a certain feature over time (30). By comparing the Akaike information criterion (AIC) and Bayesian information criterion (BIC), the model with the lowest AIC and BIC values was selected as the model with the highest fit level. A multivariable Cox proportional hazards model was used to explore the associations between the trajectories of SRH, IRH, and CMWI (2008–2014) with 4-year mortality (2014–2018). All adjustments were consistent with that in above mentioned models.

The baseline levels and longitudinal changes of SRH, IRH, and CMWI were considered as continuous variables, while their trajectories were considered as nominal variables. All analyses were performed using SAS statistical software (version 9.4; SAS Institute Inc., Cary, NC, United States). *p*-value <0.05 was considered statistically significant.

## Results

### Selection of study participants

**Figure 1:**
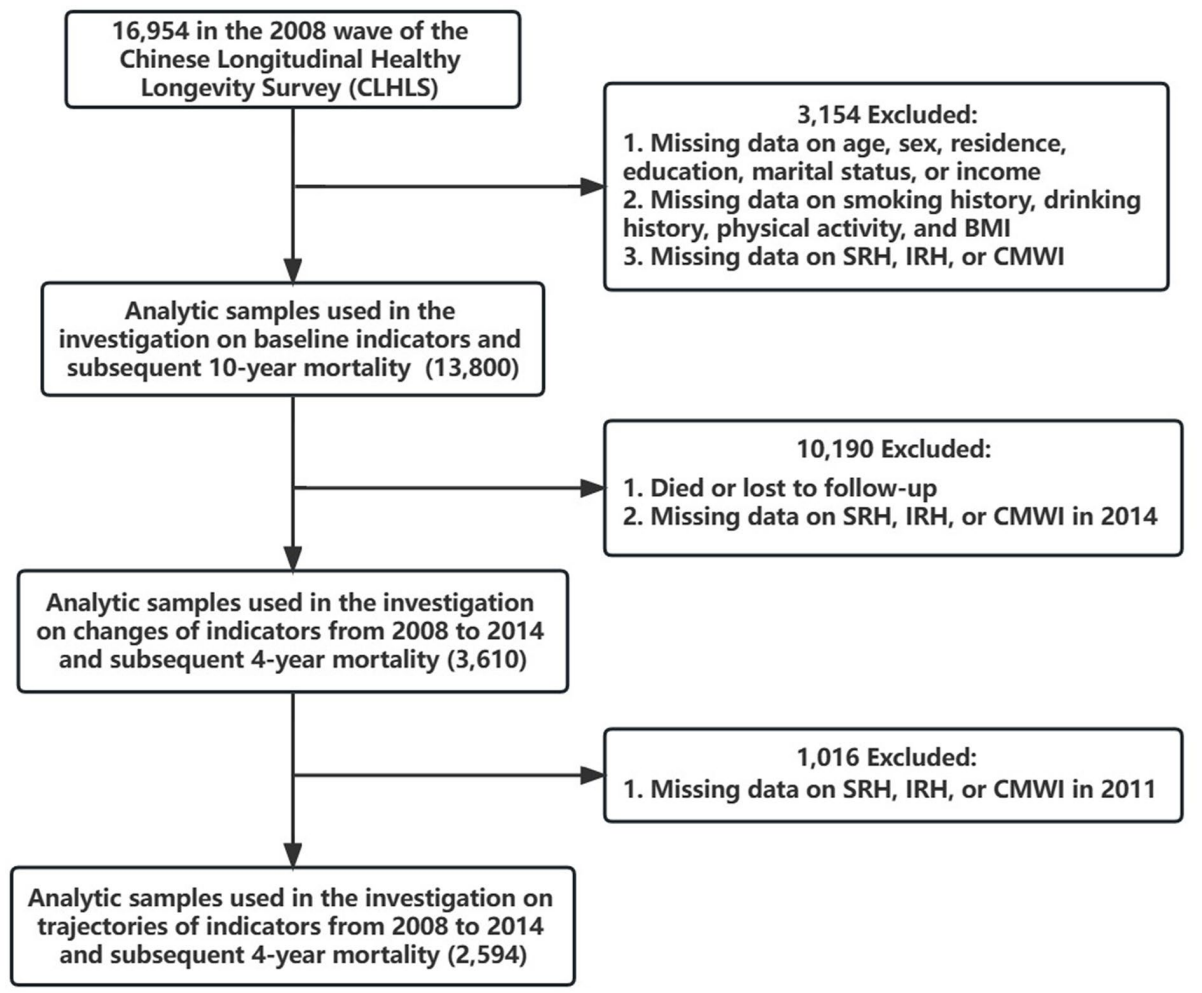
Flow chart. BMI, body mass index. SRH, self-rated health. IRH, interviewer-rated health. CMWI, Chinese multimorbidity-weighted index.

Of 16,954 participants in the 2008 wave of the CLHLS, 13,800 participants were included in the analyses of the associations between baseline indicators (SRH, IRH, or CMWI in 2008) and subsequent 10-year mortality (2008–2018), while 3,154 participants who had missing data on socio-demographic characteristics, BMI, SRH, IRH or CMWI were excluded. Then, those who died, lost to follow-up, or had missing data on SRH, IRH, or CMWI in 2014 were excluded, leaving 3,610 participants in the analyses of the associations between changes of indicators (SRH, IRH, or CMWI from 2008 to 2014) and subsequent 4-year mortality (2014–2018). Finally, since GBTM required at least three times of measurements on SRH, IRH, and CMWI, a total of 2,594 participants were included in the analyses of the associations between trajectories of indicators (SRH, IRH, or CMWI from 2008 to 2014) and subsequent 4-year mortality (2004–2018), after excluding participants who had missing data on SRH, IRH, or CMWI in 2011 (Figure 1) (31).

### Baseline SRH, IRH, and CMWI with 10-year mortality

The baseline characteristics of participants included in analyses of baseline SRH, IRH, and CMWI (2008) with 10-year mortality (2008–2018) are shown in Supplementary Table S1. Compared with participants who survived or lost to follow-up within 10 years, the older adults who died were older, with lower education level, lower annual income, smaller BMI, less physical activity. They were more likely to be in rural areas, be married or cohabitation. They also had significantly differences in SRH, IRH, and CMWI (all *p* < 0.05).

The multivariable Cox proportional hazards model showed that higher baseline SRH (HR 0.93, 95% CI 0.91–0.96), IRH (HR 0.84, 95% CI 0.81–0.87), and CMWI (HR 0.99, 95% CI 0.98–1.00) were significantly associated with lower 10-year mortality (Table 1). The results in sensitive analyses revealed consistent patterns with the main results (Supplementary Table S2). The results in males and females are also shown in Table 1. The association between CMWI (2008) and subsequent 10-year mortality (2008–2018) was not significant in females (HR 1.00, 95% CI 0.98–1.02).

**Table 1:** Baseline SRH, IRH, and CMWI with 10-year mortality from 2008 to 2018.

	Model 1		Model 2		Model 3	
	HR (95% CI)	*p*-values	HR (95% CI)	*P*-values	HR (95% CI)	*P*-values
Total population (*N* = 13,800)						
SRH	0.90 (0.88–0.93)	<0.001	0.91 (0.89–0.94)	<0.001	0.93 (0.91–0.96)	<0.001
IRH	0.70 (0.68–0.73)	<0.001	0.82 (0.79–0.85)	<0.001	0.84 (0.81–0.87)	<0.001
CMWI	1.02 (1.01–1.03)	0.001	1.00 (0.99–1.01)	0.996	0.99 (0.98–1.00)	0.020
Male (*N* = 6,294)						
SRH	0.88 (0.84–0.91)	<0.001	0.89 (0.86–0.93)	<0.001	0.92 (0.88–0.96)	<0.001
IRH	0.67 (0.64–0.71)	<0.001	0.80 (0.76–0.85)	<0.001	0.82 (0.78–0.86)	<0.001
CMWI	0.99 (0.98–1.01)	0.363	0.99 (0.97–1.00)	0.142	0.98 (0.96–0.99)	0.003
Female (*N* = 7,506)						
SRH	0.93 (0.90–0.96)	<0.001	0.93 (0.89–0.96)	<0.001	0.94 (0.91–0.98)	<0.001
IRH	0.73 (0.70–0.77)	<0.001	0.84 (0.80–0.88)	<0.001	0.85 (0.81–0.89)	<0.001
CMWI	1.04 (1.03–1.06)	<0.001	1.01 (1.00–1.03)	0.154	1.00 (0.98–1.02)	0.992

SRH, self-rated health. IRH, interviewer-rated health. CMWI, Chinese multimorbidity-weighted index. HR, hazard ratio. CI, confidence interval. Higher baseline values of SRH, IRH, and CMWI indicated better SRH, IRH, and objective health rating by CMWI. Model 1 was a crude model. Model 2 was adjusted for age and sex. Model 3 was further adjusted for residence, education, annual income, marital status, physical activity, smoking history, drinking history and body mass index based on Model 2.

### Changes of SRH, IRH, and CMWI with 4-year mortality

Since there was a considerable loss of participants after the exclusions (*n* = 10,190), we compared characteristics of study participants and excluded participants in the changes of SRH, IRH, and CMWI with 4-year mortality (Supplementary Table S3). Supplementary Table S4 shows that in analyses of changes of SRH, IRH, and CMWI (2008–2014) with 4-year mortality (2014–2018), 3,610 participants were included, in which 928 participants died (median age 84.0 [IQR 77.0–90.0]) and 2,682 participants survived or were lost to follow-up (median age 74.0 [IQR 69.0–81.0]). The older adults who died had significantly lower baseline SRH and IRH (all *p* < 0.05).

The results in Table 2 showed that participants with better changes of SRH (HR 0.93, 95% CI 0.87–0.98), IRH (HR 0.77, 95% CI 0.71–0.83), and CMWI (HR 0.97, 95% CI 0.95–0.99) showed significantly lower 4-year mortality. When stratified by sex, the results were similar in males, but in females, only the change of IRH was significantly associated with 4-year mortality (HR 0.74, 95% CI 0.66–0.84).

**Table 2:** Changes of SRH, IRH, and CMWI from 2008 to 2014 with 4-year mortality from 2014 to 2018.

	Model 1		Model 2		Model 3	
	HR (95% CI)	*P*-values	HR (95% CI)	*P*-values	HR (95% CI)	*P*-values
Total population (*N* = 3,610)						
SRH	0.94 (0.89–0.99)	0.040	0.93 (0.88–0.98)	0.013	0.93 (0.87–0.98)	0.010
IRH	0.74 (0.68–0.80)	<0.001	0.77 (0.71–0.83)	<0.001	0.77 (0.71–0.83)	<0.001
CMWI	0.99 (0.96–1.01)	0.252	0.97 (0.95–0.99)	0.010	0.97 (0.95–0.99)	0.006
Male (*N* = 1,776)						
SRH	0.90 (0.83–0.98)	0.012	0.90 (0.83–0.97)	0.006	0.89 (0.83–0.97)	0.006
IRH	0.75 (0.68–0.84)	<0.001	0.78 (0.70–0.86)	<0.001	0.78 (0.70–0.87)	<0.001
CMWI	0.97 (0.94–1.00)	0.055	0.96 (0.93–0.99)	0.008	0.96 (0.93–0.99)	0.006
Female (*N* = 1,834)						
SRH	0.99 (0.91–1.08)	0.778	0.97 (0.89–1.05)	0.456	0.96 (0.89–1.05)	0.392
IRH	0.72 (0.64–0.82)	<0.001	0.76 (0.67–0.86)	<0.001	0.74 (0.66–0.84)	<0.001
CMWI	1.01 (0.97–1.04)	0.633	0.98 (0.95–1.02)	0.386	0.98 (0.94–1.02)	0.296

SRH, self-rated health. IRH, interviewer-rated health. CMWI, Chinese multimorbidity-weighted index. HR, hazard ratio. CI, confidence interval. Higher values of SRH, IRH, and CMWI changes indicated better changes in SRH, IRH, and objective health rating by CMWI. Model 1 was a crude model. Model 2 was adjusted for age and sex. Model 3 was further adjusted for residence, education, annual income, marital status, physical activity, smoking history, drinking history and body mass index based on Model 2.

### Trajectories of SRH, IRH, and CMWI with 4-year mortality

The baseline characteristics of participants included in analyses on the trajectory of predictors and 4-year mortality from 2014 to 2018 was shown in Supplementary Table S5. Compared with participants who survived or were lost to follow-up within 4 years, the older adults who died had significantly lower SRH and IRH (all *p* < 0.05). Supplementary Table S6 indicated that when trajectories were divided into 2, the models of SRH, IRH, and CMWI had the lowest AIC and BIC values (AIC = −11345.1, BIC = −11368.5 for the trajectory of SRH; AIC = −8887.4, BIC = −8910.8 for the trajectory of IRH; AIC = −16987.7, BIC = −17011.1 for the trajectory of CMWI). Supplementary Figure S1 showed the trajectories of SRH, IRH, and CMWI, respectively. The higher SRH, IRH, and CMWI trajectories were named “high SRH/IRH/CMWI,” and the others were named “low and declining SRH/IRH/CMWI.”

Taking “low and declining SRH/IRH/CMWI” as the reference, we found that high SRH (HR 0.58, 95% CI 0.48–0.70), high IRH (HR 0.66, 95% CI 0.55–0.80), and high CMWI (HR 0.74, 95% CI 0.61–0.89) were associated with lower 4-year mortality (Table 3). However, the association between the trajectory of CMWI and 4-year mortality was not significant in females (HR 0.76, 95% CI 0.57–1.02).

**Table 3:** Trajectories of SRH, IRH, and CMWI from 2008 to 2014 with 4-year mortality from 2014 to 2018.

	Model 1		Model 2		Model 3	
	HR (95% CI)	*P*-values	HR (95% CI)	*P*-values	HR (95% CI)	*P*-values
Total population (*N* = 2,594)						
Low and declining SRH (*N* = 383)	Reference	/	Reference	/	Reference	/
High SRH (*N* = 2,211)	0.65 (0.54–0.79)	<0.001	0.57 (0.47–0.69)	<0.001	0.58 (0.48–0.70)	<0.001
Low and declining IRH (*N* = 1,774)	Reference	/	Reference	/	Reference	/
High IRH (*N* = 820)	0.57 (0.47–0.69)	<0.001	0.65 (0.54–0.79)	<0.001	0.66 (0.55–0.80)	<0.001
Low and declining CMWI (*N* = 2,307)	Reference	/	Reference	/	Reference	/
High CMWI (*N* = 287)	0.99 (0.83–1.19)	0.931	0.80 (0.67–0.97)	0.021	0.74 (0.61–0.89)	0.002
Male (*N* = 1,298)						
Low and declining SRH (*N* = 147)	Reference	/	Reference	/	Reference	/
High SRH (*N* = 1,151)	0.50 (0.39–0.66)	<0.001	0.49 (0.37–0.64)	<0.001	0.51 (0.39–0.67)	<0.001
Low and declining IRH (*N* = 836)	Reference	/	Reference	/	Reference	/
High IRH (*N* = 462)	0.55 (0.43–0.70)	<0.001	0.64 (0.50–0.82)	<0.001	0.66 (0.51–0.84)	<0.001
Low and declining CMWI (*N* = 1,159)	Reference	/	Reference	/	Reference	/
High CMWI (*N* = 139)	0.86 (0.68–1.10)	0.243	0.79 (0.62–1.01)	0.0624	0.72 (0.57–0.94)	0.014
Female (*N* = 1,296)						
Low and declining SRH (*N* = 236)	Reference	/	Reference	/	Reference	/
High SRH (*N* = 1,060)	0.76 (0.58–1.00)	0.0521	0.65 (0.49–0.86)	0.002	0.68 (0.51–0.90)	0.006
Low and declining IRH (*N* = 938)	Reference	/	Reference	/	Reference	/
High IRH (*N* = 358)	0.57 (0.42–0.76)	<0.001	0.67 (0.50–0.90)	0.008	0.68 (0.51–0.92)	0.013
Low and declining CMWI (*N* = 1,148)	Reference	/	Reference	/	Reference	/
High CMWI (*N* = 148)	1.14 (0.86–1.50)	0.361	0.81 (0.61–1.08)	0.152	0.76 (0.57–1.02)	0.067

SRH, self-rated health. IRH, interviewer-rated health. CMWI, Chinese multimorbidity-weighted index. HR, hazard ratio. CI, confidence interval. Model 1 was a crude model. Model 2 was adjusted for age and sex. Model 3 was further adjusted for residence, education, annual income, marital status, physical activity, smoking history, drinking history and body mass index based on Model 2.

## Discussion

Our study found that baseline SRH, IRH, and CMWI were all negatively associated with 10-year mortality in Chinese older adults. Better changes and trajectories of SRH, IRH, and CMWI were also significantly associated with lower 4-year mortality in Chinese older adults.

Previous studies have revealed the negative associations of baseline SRH, IRH, and objective health with mortality, which were in line with our results (11, 16, 29, 32, 33). First, SRH is a subjective assessment based on objective health status (34), therefore serves as a statistical indicator with a condensed overview of physical information (5). Second, compared to objective indicators of health, SRH might capture the burden of subclinical conditions, such as high blood pressure, early stages of diabetes, and preclinical forms of cancer (35). Third, poor SRH sometimes mirrors mental stress, which can lead to elevations in serum proinflammatory cytokines (36–38). Inflammation is associated with symptoms of sickness behavior, such as lethargy, decreased appetite, and behavioral withdrawal, which in turn will have an impact on SRH (39). Additionally, the dysregulation of inflammation leads to the development of illnesses and raises the risk of mortality in older adults (40–42). Consequently, elevated levels of circulating inflammatory markers contribute to both a decrease in SRH and an increase in mortality risk (43).

Some recent studies reported that IRH is a better indicator of mortality than SRH, and investigated the potential mechanisms underlying the association. For instance, using data from CLHLS, Feng et al. found that the association between IRH and mortality remained significant even after adjusting for SRH (7). Similarly, Todd M A and Goldman N used an older adults cohort in Taiwan to compare the predictive power of SRH, IRH, and health ratings from physicians on 5-year mortality, and revealed that IRH was the most accurate predictor (16). Several reasons have been put forward to explain why IRH may be a better predictor of mortality than SRH. First, when evaluating SRH, respondents may underestimate health-related behaviors and functional limitations (44), whereas interviewers who assess IRH can weigh and integrate individual information more effectively (45). Moreover, interviewers may be able to capture unmeasured health information that SRH lacks through on-site observations, such as respondents’ appearance and living situation. Second, in CLHLS, the rating process is done immediately following the interviewer’s completion of data collection, therefore IRH incorporates detailed information about the participants overall health, including their SRH. Third, trained interviewers can maintain a consistent and relatively objective evaluation standard when investigating multiple respondents, avoiding personal biases to some extent. Furthermore, interviewers generally have higher educational attainment than the respondents, which could help them accurately summarize information during the interview and provide a more accurate assessment of the respondent’s health conditions (7, 16, 44). Incorporating both self-rated and interviewer-rated health could provide a more complete understanding of a person’s health status.

Previous studies produced contradictory results of gender differences in the association between SRH and mortality (46). Our results indicate that SRH was associated with mortality in male. In females, however, there was no association between the change of SRH and mortality. These sex differences in the associations between subjective health indicators and mortality may result from the different health self-perceptions, which means the meaning of “health” differs between sexes (46). Also, the covariates may be different between males and females. Besides, it is noteworthy that sex-stratified analyses showed that compared to male, the association between CMWI and mortality was not significant in females, which was consistent with result from Wuorela et al. (47). One explanation could be that although women have poorer health status, they can tolerate more frailty than men. It could also result from biological and social factors (48, 49).

Multimorbidity, defined as the presence of two or more chronic diseases in the same individual, reflects worse health status and is associated with mortality (50). Previous study has shown that CMWI is a superior predictor of mortality among the Chinese older adults compared to other multimorbidity indexes, as its disease weights were tailored to the Chinese middle-aged and older community-dwelling adults (11). When stratified by sex, we found that baseline CMWI, together with its change and trajectory was not associated with subsequent mortality in female. This may be due to several limitations. First, mental function was not considered in the development and validation of CMWI due to limited mental health measures. This may result in information bias, as there are sex-differences in mental health conditions. Second, the collection of information on chronic diseases were largely gathered by self-report, which may lead to reporting bias. Third, the burden of multimorbidity may have been underestimated because the severity of the disease was not taken into account while calculating CMWI. Thus, further studies are needed to investigate the sex-specific factors behind CMWI and explore a more universally applicable indicator of mortality for both males and females.

Previous studies have investigated the association between changes and trajectories in SRH and mortality, but the results have not been consistent. For instance, the Women’s Health and Aging Study suggested that change in SRH was a stronger indicator of mortality than baseline SRH (23). While another study found that SRH trajectories did not have a significant, direct impact on mortality (24). The inconsistent findings might stem from variations in investigation date, sample size, studying setting, and other factors. Our study confirmed significant associations of changes and trajectories in SRH with mortality, and further indicated that the changes and trajectories of IRH and CMWI were also associated with mortality in Chinese older adults. These indicators are dynamic and change over time during the follow-up period (46, 51). Neglecting to assess SRH as a dynamic indicator may lead to a misinterpretation of the association between SRH and mortality (52). One study revealed that baseline SRH was only associated with 4-year mortality, but not with 9-year mortality among old female (53). A research also indicated that poor baseline SRH was associated with higher risk of 10-year mortality, but no longer with 18-year mortality in older male (38). Idler and Benyamini proposed the *trajectory hypothesis*, which suggested that people incorporated changes in prior health status into their current health evaluations, and that poor SRH indicated assessments of their future decline or death (24, 29). Since the changes and trajectories are originated from different interview points, their evaluation often involve all the information of multiple time points. Besides, changes in other information such as socioeconomics factors, which are not included in the questionnaire can be reflected in the changes and trajectories of indicators (7). Therefore, the changes and trajectories of indicators contain a wider range of information than baseline.

Numerous studies have explored a range of mortality indicators, such as multimorbidity-weighted index and the Elixhauser comorbidity index. However, their widespread use in densely populated developing countries like China is challenging due to their high cost and implementation complexity (11). Besides, previous studies have also shown that the combination of subjective and objective health indicators could provide a simple measure for risk classification (19). Our study highlights the applicability of SRH, IRH, and CMWI, particularly in geriatric health-related studies. Because SRH, IRH, and CMWI are easy to use and cost-effective, it is realistic to popularize them in grassroots communities and rural areas in China, and conducive to the government’s preliminary understanding of the health status of the local older adults. Formulating a more appropriate health promotion plan for the older adults can promote the healthy aging construction of Chinese society.

## Strengths and limitations

To the best of our knowledge, this study was the first prospective cohort study to comprehensively explore the association of baseline SRH, IRH, and CMWI, their changes and trajectories with subsequent mortality in Chinese older adults. Moreover, with the national sample, the CLHLS represented community-based older adults from 22 Chinese provinces, ensuring that our findings were reliable and generalizable.

This study had several limitations. First, we merged “poor” and “very poor” SRH due to the limitations of sample size, which may lead to information bias. Also, since we used the continuous forms of SRH and IRH, the results of baseline values and changes of SRH and IRH with mortality might be difficult to interpret. However, the sample sizes in some groups were relatively small when considering SRH and IRH as categorial variables. We have conducted sensitive analyses to investigate the associations of SRH and IRH as categorical variables with 10-year mortality. The results were consistent with the main findings, supporting the robustness of our approach. Second, the number of participants included in the analysis of the trajectory was relatively small, thus, the trajectory of IRH was not smooth enough. However, this limitation did not influence the association between trajectories of indicators and mortality. Further studies are needed in a larger population. Third, some of the covariates, such as physical activity and smoking history, were lacking more detailed data in the questionnaire of CLHLS, which may result in potential residual confounding in the associations. Finally, some potential covariates, such as type of work, social participation, may have been neglected in the adjustment.

## Conclusion

SRH, IRH, and CMWI are practical and accurate indicators of mortality in the Chinese older adults. More researches are required to promote the application and generalization of cost-effective indicators in the field of health assessment and monitoring among Chinese older adults.

## Data availability statement

The original contributions presented in the study are included in the article/Supplementary material, further inquiries can be directed to the corresponding authors.

## Author contributions

PS designed the study. ZR and SS managed and analyzed the data. SS and SC prepared the first draft. SS, JC, SC, KT, SL, WS, LH, and QY reviewed and edited the manuscript, with comments from PS. All authors involved in revising the manuscript, had full access to the data, and gave final approval of the submitted versions.

## Funding

Data used for this research was provided by the study entitled “Chinese Longitudinal Healthy Longevity Survey” (CLHLS) managed by the Center for Healthy Aging and Development Studies, Peking University. CLHLS was supported by funds from the U.S. National Institutes on Aging (NIA), China Natural Science Foundation, China Social Science Foundation, and UNFPA. The study sponsors had no role in the study design, analysis, interpretation of data, writing of the report, or in the decision to submit the paper for publication.

## Conflict of interest

The authors declare that the research was conducted in the absence of any commercial or financial relationships that could be construed as a potential conflict of interest.

## Publisher’s note

All claims expressed in this article are solely those of the authors and do not necessarily represent those of their affiliated organizations, or those of the publisher, the editors and the reviewers. Any product that may be evaluated in this article, or claim that may be made by its manufacturer, is not guaranteed or endorsed by the publisher.

## References

[1] Liangchu Y. Research on fertility policy to cope with the challenge of population aging. Soc Sec Stud. (2021) 78, 05:39–48. doi: 10.3969/j.issn.1674-4802.2021.05.004

[2] Tu WJ Zeng X Liu Q. Aging tsunami coming: the main finding from China’s seventh national population census. Aging Clin Exp Res. (2022) 34:1159–63. doi: 10.1007/s40520-021-02017-4, PMID: 34727357

[3] Fang EF Scheibye-Knudsen M Jahn HJ Li J Ling L Guo H. A research agenda for aging in China in the 21st century. Ageing Res Rev. (2015) 24:197–205. doi: 10.1016/j.arr.2015.08.003, PMID: 26304837 PMC5179143

[4] Guixin W. Future challenges and coping strategies of population ageing in China. Governance. (2022) 10:50–6.

[5] Jylhä M. What is self-rated health and why does it predict mortality? Towards a unified conceptual model. Soc Sci Med. (2009) 69:307–16. doi: 10.1016/j.socscimed.2009.05.013, PMID: 19520474

[6] Mossey JM Shapiro EJAJOPH. Self-rated health: a predictor of mortality among the elderly. Am J Public Health. (1982) 72:800–8. doi: 10.2105/AJPH.72.8.800 7091475 PMC1650365

[7] Feng Q Zhu H Zhen Z Gu D. Self-rated health, interviewer-rated health, and their predictive powers on mortality in old age. J Gerontol B Psychol Sci Soc Sci. (2016) 71:538–50. doi: 10.1093/geronb/gbu186, PMID: 25617400 PMC6366535

[8] De Bruin A. Health interview surveys: towards international harmonization of methods and instruments. WHO Reg Publ Eur Ser. (1996) 58:1–161. 8857196

[9] Zhu H Feng Q Gu DJIJOPS. Self-rated health and interviewer-rated health: differentials in predictive power for mortality among subgroups of Chinese elders. Int J Popul Stud. (2017) 2:74–91. doi: 10.18063/IJPS.2016.02.007

[10] Lima-Costa MF Cesar CC Chor D Proietti FA. Self-rated health compared with objectively measured health status as a tool for mortality risk screening in older adults: 10-year follow-up of the Bambuí cohort study of aging. Am J Epidemiol. (2012) 175:228–35. doi: 10.1093/aje/kwr290, PMID: 22193172

[11] Hu WH Liu YY Yang CH Zhou T Yang C Lai YS. Developing and validating a Chinese multimorbidity-weighted index for middle-aged and older community-dwelling individuals. Age Ageing. (2022) 51:afab274. doi: 10.1093/ageing/afab274, PMID: 35211718

[12] Garbarski D Schaeffer NC Dykema J. Interviewers’ ratings of respondents’ health: predictors and association with mortality. J Gerontol B Psychol Sci Soc Sci. (2019) 74:1213–21. doi: 10.1093/geronb/gbx146, PMID: 29220523 PMC6748795

[13] Idler EL Benyamini YJJOH Behavior S. Self-rated health and mortality: a review of twenty-seven community studies. J. Health Soc. Behav. (1997) 38:21–37. 9097506

[14] Idler EL Kasl S. Health perceptions and survival: do global evaluations of health status really predict mortality? J Gerontol. (1991) 46:S55–65. doi: 10.1093/geronj/46.2.S55, PMID: 1997583

[15] Miller AC O’reilly D Kee F Cruise S Young I. Multimorbidity, activity limitation and self-reported health all predict mortality risk, but better measures were required. J Clin Epidemiol. (2022) 144:144–62. doi: 10.1016/j.jclinepi.2021.12.010, PMID: 34910980

[16] Todd MA Goldman N. Do interviewer and physician health ratings predict mortality? A comparison with self-rated health. Epidemiology. (2013) 24:913–20. doi: 10.1097/EDE.0b013e3182a713a8, PMID: 24045721 PMC3968811

[17] Valanis BG Yeaworth RJRIN Health. Ratings of physical and mental health in the older bereaved. Res Nurs Health. (1982) 5:137–46. doi: 10.1002/nur.4770050305 6923442

[18] Yuzhi L Qiang L. The relationship between self-rated health and mortality risk of the Chinese oldest old. Chin J Popul Sci. (2004) 04:30–37+81. doi: 10.3969/j.issn.1000-7881.2004.04.004

[19] Mutz J Lewis CM. Cross-classification between self-rated health and health status: longitudinal analyses of all-cause mortality and leading causes of death in the UK. Sci Rep. (2022) 12:459. doi: 10.1038/s41598-021-04016-x, PMID: 35013388 PMC8748682

[20] Fayers PM Sprangers MAG. Understanding self-rated Health. Lancet. (2002) 359:187–8. doi: 10.1016/S0140-6736(02)07466-4 11812551

[21] Feenstra M Van Munster BC Macneil Vroomen JL De Rooij SE Smidt N. Trajectories of self-rated health in an older general population and their determinants: the lifelines cohort study. BMJ Open. (2020) 10:E035012. doi: 10.1136/bmjopen-2019-035012, PMID: 32075843 PMC7045095

[22] Ferraro KF Kelley-Moore JA. Self-rated health and mortality among black and white adults: examining the dynamic evaluation thesis. J Gerontol B Psychol Sci Soc Sci. (2001) 56:S195–205. doi: 10.1093/geronb/56.4.S195, PMID: 11445612

[23] Han B Phillips C Ferrucci L Bandeen-Roche K Jylha M Kasper J. Change in self-rated health and mortality among community-dwelling disabled older women. Gerontologist. (2005) 45:216–21. doi: 10.1093/geront/45.2.216, PMID: 15799986

[24] Miller TR Wolinsky FD. Self-rated health trajectories and mortality among older adults. J Gerontol B Psychol Sci Soc Sci. (2007) 62:S22–7. doi: 10.1093/geronb/62.1.S22, PMID: 17284562 PMC2104470

[25] Lv X Li W Ma Y Chen H Zeng Y Yu X. Cognitive decline and mortality among community-dwelling Chinese older people. BMC Med. (2019) 17:63. doi: 10.1186/s12916-019-1295-8, PMID: 30871536 PMC6419492

[26] Lv YB Gao X Yin ZX Chen HS Luo JS Brasher MS. Revisiting the association of blood pressure with mortality in oldest old people in China: community based, longitudinal prospective study. BMJ. (2018) 361:K2158. doi: 10.1136/bmj.k2158 29871897 PMC5987177

[27] Zeng Y. Towards deeper research and better policy for healthy aging --using the unique data of Chinese longitudinal healthy longevity survey. China Econo J. (2012) 5:131–49. doi: 10.1080/17538963.2013.764677, PMID: 24443653 PMC3893304

[28] Zeng Y Feng Q Hesketh T Christensen K Vaupel JW. Survival, disabilities in activities of daily living, and physical and cognitive functioning among the oldest-old in China: a cohort study. Lancet. (2017) 389:1619–29. doi: 10.1016/S0140-6736(17)30548-2, PMID: 28285816 PMC5406246

[29] Benyamini Y Idler ELJROA. Community studies reporting association between self-rated health and mortality: additional studies, 1995 to 1998. Res. Aging. (1999) 21:392–401. doi: 10.1177/0164027599213002

[30] Feldman B Shen J Chen C Shi J Xiang H. Perceived health after adult traumatic brain injury: a Group-Based Trajectory Modeling (GBTM) analysis. Brain Inj. (2020) 34:741–50. doi: 10.1080/02699052.2020.1753111, PMID: 32320317

[31] Nguena Nguefack HL Pagé MG Katz J Choinière M Vanasse A Dorais M. Trajectory modelling techniques useful to epidemiological research: a comparative narrative review of approaches. Clin Epidemiol. (2020) 12:1205–22. doi: 10.2147/CLEP.S265287, PMID: 33154677 PMC7608582

[32] Larsson D Hemmingsson T Allebeck P Lundberg I. Self-rated health and mortality among young men: what is the relation and how may it be explained? Scand J Public Health. (2002) 30:259–66. doi: 10.1080/14034940210133997 12680501

[33] Moreno X Huerta M Albala C. Global self-rated health and mortality in older people. Gac Sanit. (2014) 28:246–52. doi: 10.1016/j.gaceta.2013.07.006, PMID: 24359681

[34] Fan Y He D. Self-rated health, socioeconomic status and all-cause mortality in Chinese middle-aged and elderly adults. Sci Rep. (2022) 12:1–8. doi: 10.1038/s41598-022-13502-9 35662273 PMC9166789

[35] Benyamini Y. Why does self-rated health predict mortality? An update on current knowledge and a research agenda for psychologists. Psychol Health. (2011) 26:1407–13. doi: 10.1080/08870446.2011.621703, PMID: 22111660

[36] Benyamini Y Idler EL Leventhal H Leventhal EAJTJOGSBPS Sciences S. Positive affect and function as influences on self-assessments of health: expanding our view beyond illness and disability. J Gerontol B Psychol Sci Soc Sci. (2000) 55:P107–16. doi: 10.1093/geronb/55.2.P107 10794189

[37] Brumpton BM Leivseth L Romundstad PR Langhammer A Chen Y Camargo CA Jr. The joint association of anxiety, depression and obesity with incident asthma in adults: the hunt study. Int J Epidemiol. (2013) 42:1455–63. doi: 10.1093/ije/dyt151 24008330

[38] Lyyra T-M Heikkinen E Lyyra A-L Jylhä M. Self-rated health and mortality: could clinical and performance-based measures of health and functioning explain the association? Arch Gerontol Geriatr. (2006) 42:277–88. doi: 10.1016/j.archger.2005.08.001, PMID: 16214245

[39] Dantzer R Kelley KW. Twenty years of research on cytokine-induced sickness behavior. Brain Behav Immun. (2007) 21:153–60. doi: 10.1016/j.bbi.2006.09.006 17088043 PMC1850954

[40] Allin KH Bojesen SE Nordestgaard BG. Baseline C-reactive protein is associated with incident cancer and survival in patients with cancer. J Clin Oncol. (2009) 27:2217–24. doi: 10.1200/JCO.2008.19.8440 19289618

[41] Black PH. Stress and the inflammatory response: a review of neurogenic inflammation. Brain Behav Immun. (2002) 16:622–53. doi: 10.1016/S0889-1591(02)00021-1 12480495

[42] Roubenoff R Parise H Payette HA Abad LW D’agostino R Jacques PF. Cytokines, insulin-like growth factor 1, sarcopenia, and mortality in very old community-dwelling men and women: the Framingham Heart Study. Am J Med. (2003) 115:429–35. doi: 10.1016/j.amjmed.2003.05.001 14563498

[43] Christian LM Glaser R Porter K Malarkey WB Beversdorf D Kiecolt-Glaser JK. Poorer self-rated health is associated with elevated inflammatory markers among older adults. Psychoneuroendocrinology. (2011) 36:1495–504. doi: 10.1016/j.psyneuen.2011.04.003, PMID: 21601365 PMC3161147

[44] Smith KV Goldman N. Measuring health status: self-, interviewer, and physician reports of overall health. J Aging Health. (2011) 23:242–66. doi: 10.1177/0898264310383421, PMID: 21041293 PMC3727648

[45] Brissette I Leventhal H Leventhal EA. Observer ratings of health and sickness: can other people tell us anything about our health that we don’t already know? Health Psychol. (2003) 22:471–8. doi: 10.1037/0278-6133.22.5.471, PMID: 14570530

[46] Lyyra T-M Leskinen E Jylhä M Heikkinen E. Self-rated health and mortality in older men and women: a time-dependent covariate analysis. Arch Gerontol Geriatr. (2009) 48:14–8. doi: 10.1016/j.archger.2007.09.004, PMID: 17950481

[47] Wuorela M Lavonius S Salminen M Vahlberg T Viitanen M Viikari L. Self-rated health and objective health status as predictors of all-cause mortality among older people: a prospective study with a 5-, 10-, and 27-year follow-up. BMC Geriatr. (2020) 20:120. doi: 10.1186/s12877-020-01516-9, PMID: 32228464 PMC7106830

[48] Gordon EH Peel NM Samanta M Theou O Howlett SE Hubbard RE. Sex differences in frailty: a systematic review and meta-analysis. Exp Gerontol. (2017) 89:30–40. doi: 10.1016/j.exger.2016.12.021, PMID: 28043934

[49] Mutz J Roscoe CJ Lewis CM. Exploring health in the UK Biobank: associations with sociodemographic characteristics, psychosocial factors, lifestyle and environmental exposures. BMC Med. (2021) 19:240. doi: 10.1186/s12916-021-02097-z 34629060 PMC8504077

[50] Rizzuto D Melis RJ Angleman S Qiu C Marengoni A. Effect of chronic diseases and multimorbidity on survival and functioning in elderly adults. J Am Geriatr Soc. (2017) 65:1056–60. doi: 10.1111/jgs.14868 28306158

[51] Strawbridge WJ Wallhagen MI. Self-rated health and mortality over three decades: results from a time-dependent covariate analysis. Res Aging. (1999) 21:402–16.

[52] Nery Guimarães JM Chor D Werneck GL Carvalho MS Coeli CM Lopes CS. Association between self-rated health and mortality: 10 years follow-up to the Pró-Saúde cohort study. BMC Public Health. (2012) 12:1–10. doi: 10.1186/1471-2458-12-676 22905737 PMC3491020

[53] Benyamini Y Blumstein T Lusky A Modan BJTG. Gender differences in the self-rated health–mortality association: is it poor self-rated health that predicts mortality or excellent self-rated health that predicts survival? Gerontologist. (2003) 43:396–405. doi: 10.1093/geront/43.3.396 12810904

