# Supplementary material for: Self-rated health, interviewer-rated health, and objective health, their changes and trajectories over time, and the risk of mortality in Chinese adults
Source: Front Public Health. 2023 Jun 20;11:1137527. doi: 10.3389/fpubh.2023.1137527 (PMC10318337; doi:10.3389/fpubh.2023.1137527)
Supplement: Supplementary file 1 [file Data_Sheet_1.docx]

Contents

[Table S1. Characteristics of participants included in analyses of 10-year mortality from 2008 to 2018 1](#_Toc133101165)

[Table S2. Sensitive analyses on the associations of SRH and IRH with 10-year mortality from 2008 to 2018 4](#_Toc133101166)

[Table S3. Characteristics of included participants and excluded participants in the changes of SRH, IRH, and CMWI with 4-year mortality 5](#_Toc133101167)

[Table S4. Characteristics of participants included in analyses of changes of indicators with 4-year mortality from 2014 to 2018 7](#_Toc133101168)

[Table S5. Characteristics of participants included in analyses of trajectories of indicators with 4-year mortality from 2014 to 2018 9](#_Toc133101169)

[Table S6. Trajectory groups 11](#_Toc133101170)

[Figure S1. Trajectories of SRH, IRH, and CMWI 12](#_Toc133101171)

# Table S1. Characteristics of participants included in analyses of 10-year mortality from 2008 to 2018

| **Baseline Characteristics** | | **Died (*N*=7,506)** | **Survived or lost to follow-up (*N*=6,294)** | ***P*-value** |
| --- | --- | --- | --- | --- |
| Age, year | | 90.0 (84.0-97.0) | 79.0 (70.0-89.0) | <0.001 |
| Sex | |  |  | 0.107 |
|  | Male | 3,376 (45.0) | 2,918 (46.4) |  |
|  | Female | 4,130 (55.0) | 3,376 (53.6) |  |
| Residence | | |  | <0.001 |
|  | Rural | 4,819 (64.2) | 3,408 (54.2) |  |
|  | Urban | 2,687 (35.8) | 2,886 (45.9) |  |
| Education | | |  | <0.001 |
|  | Illiteracy | 4,997 (66.6) | 3,138 (49.9) |  |
|  | Primary education | 1,953 (26.0) | 2,160 (34.3) |  |
|  | Middle school or above | 556 (7.4) | 996 (15.8) |  |
| Annual income, yuan | | | | <0.001 |
|  | 0-29,999 | 5,696 (75.9) | 4,486 (71.3) |  |
|  | 30000-59,999 | 1,045 (13.9) | 979 (15.6) |  |
|  | > 60,000 | 765 (10.2) | 829 (13.2) |  |
| Marital status | | | | <0.001 |
|  | Married or cohabitation | 5,691 (75.8) | 3,347 (53.2) |  |
|  | Unmarried or widowed | 1,815 (24.2) | 2,947 (46.8) |  |
| Physical activity | |  |  | <0.001 |
|  | No | 5,645 (75.2) | 4,041 (64.2) |  |
|  | Yes | 1,861 (24.8) | 2,253 (35.8) |  |
| Smoking history | | |  | 0.289 |
|  | Never smoking | 4,881 (65.0) | 4,148 (65.9) |  |
|  | Ever smoking | 2,625 (35.0) | 2,146 (34.1) |  |
| Drinking history | | |  | 0.102 |
|  | Never drinking | 5,088 (67.8) | 4,349 (69.1) |  |
|  | Ever drinking | 2,418 (32.2) | 1,945 (30.9) |  |
| BMI, kg/m^2^ | | 19.5 (17.6-21.9) | 20.8 (18.7-23.3) | <0.001 |
| SRH | |  |  | <0.001 |
|  | Poor or very poor | 1,296 (17.3) | 849 (13.5) |  |
|  | Fair | 2,554 (34.0) | 2,170 (34.5) |  |
|  | Good | 2,880 (38.4) | 2,441 (38.8) |  |
|  | Very good | 776 (10.3) | 834 (13.3) |  |
| IRH | |  |  | <0.001 |
|  | Moderately or severely ill | 111 (1.5) | 60 (1.0) |  |
|  | Slightly ill | 1,171 (15.6) | 536 (8.5) |  |
|  | Relative healthy | 4,768 (63.5) | 3,801 (60.4) |  |
|  | Healthy | 1,456 (19.4) | 1,897 (30.1) |  |
| CMWI | | -1.5 ([-3.2] - [-0.2]) | -1.5 ([-3.6] - [-0.2]) | 0.001 |

Notes: Values are presented as number (N) with percent (%) or medians with interquartile ranges (IQRs). BMI, body mass index. SRH, self-rated health. IRH, interviewer-rated health. CMWI, Chinese multimorbidity-weighted index.

# Table S2. Sensitive analyses on the associations of SRH and IRH with 10-year mortality from 2008 to 2018

|  | | **Model 1** | |  | **Model 2** | |  | **Model 3** | |
| --- | --- | --- | --- | --- | --- | --- | --- | --- | --- |
|  |  | **HR (95% CI)** | ***P*-value** |  | **HR (95% CI)** | ***P*-value** |  | **HR (95% CI)** | ***P*-value** |
| **SRH** | |  |  |  |  |  |  |  |  |
|  | Poor or very poor (*N*=2,145) | Reference | / |  | Reference | / |  | Reference | / |
|  | Fair (*N*=4,724) | 0.83 (0.77-0.88) | <0.001 |  | 0.76 (0.71-0.81) | <0.001 |  | 0.79 (0.74-0.84) | <0.001 |
|  | Good (*N*=5,321) | 0.82 (0.76-0.87) | <0.001 |  | 0.77 (0.72-0.82) | <0.001 |  | 0.81 (0.75-0.86) | <0.001 |
|  | Very good (*N*=1,610) | 0.69 (0.63-0.75) | <0.001 |  | 0.71 (0.65-0.77) | <0.001 |  | 0.78 (0.71-0.85) | <0.001 |
| **IRH** | |  |  |  |  |  |  |  |  |
|  | Moderately or severely ill (*N*=171) | Reference | / |  | Reference | / |  | Reference | / |
|  | Slightly ill (*N*=1,707) | 1.06 (0.87-1.29) | 0.559 |  | 0.93 (0.77-1.13) | 0.480 |  | 0.92 (0.76-1.12) | 0.424 |
|  | Relative healthy (*N*=8,569) | 0.71 (0.59-0.86) | <0.001 |  | 0.73 (0.61-0.89) | 0.001 |  | 0.73 (0.61-0.89) | 0.001 |
|  | Healthy (*N*=3,353) | 0.49 (0.40-0.59) | <0.001 |  | 0.62 (0.51-0.75) | <0.001 |  | 0.64 (0.52-0.77) | <0.001 |

Notes: SRH, self-rated health. IRH, interviewer-rated health. HR, hazard ratio. CI, confidence interval. Model 1 was a crude model. Model 2 was adjusted for age and sex. Model 3 was further adjusted for residence, education, annual income, marital status, physical activity, smoking history, drinking history and body mass index based on Model 2.

# **Table S3.** **Characteristics of included participants and excluded participants in the changes of SRH, IRH, and CMWI with 4-year mortality**

| **Baseline Characteristics** | | **Included (*N*=3,610)** | **Excluded (*N*=10,190)** | ***P*-value** |
| --- | --- | --- | --- | --- |
| Age, year | | 76.5 (70.0-84.0) | 90.0 (81.0-97.0) | <0.001 |
| Sex | |  |  | <0.001 |
|  | Male | 1,776 (49.2) | 4,518 (44.3) |  |
|  | Female | 1,834 (50.8) | 5,762 (55.7) |  |
| Residence | | |  | <0.001 |
|  | Rural | 2,255 (62.5) | 5,972 (58.6) |  |
|  | Urban | 1,355 (37.5) | 4,218 (41.4) |  |
| Education, years | | |  | <0.001 |
|  | Illiteracy | 1,773 (49.1) | 6,362 (62.4) |  |
|  | Primary education | 1,316 (36.5) | 2,797 (27.5) |  |
|  | Middle school or above | 521 (14.4) | 1,031 (10.1) |  |
| Annual income, yuan | | | | <0.001 |
|  | 0-29,999 | 2,812 (77.9) | 7,370 (72.3) |  |
|  | 30,000-59,999 | 468 (13.0) | 1,556 (15.3) |  |
|  | > 60,000 | 330 (9.1) | 1,264 (12.4) |  |
| Marital status | | | | <0.001 |
|  | Married or cohabitation | 1,672 (46.3) | 7,366 (72.3) |  |
|  | Unmarried or widowed | 1,938 (53.7) | 2,824 (27.7) |  |
| Physical activity | |  |  | <0.001 |
|  | No | 2,271 (62.9) | 7,415 (72.8) |  |
|  | Yes | 1,339 (37.1) | 2,775 (27.2) |  |
| Smoking history | | |  | <0.001 |
|  | Never smoking | 2,228 (61.7) | 6,801 (66.7) |  |
|  | Ever smoking | 1,382 (38.3) | 3,389 (33.3) |  |
| Drinking history | | |  | <0.001 |
|  | Never drinking | 2,330 (64.5) | 7,107 (69.7) |  |
|  | Ever drinking | 1,280 (35.5) | 3,083 (30.3) |  |
| BMI, kg/m^2^ | | 20.8 (18.8-23.4) | 19.8 (17.8-22.2) | <0.001 |
| SRH | |  |  | <0.001 |
|  | Poor or very poor | 487 (13.5) | 1,658 (16.3) |  |
|  | Fair | 1,129 (31.3) | 3,595 (35.3) |  |
|  | Good | 1,480 (41.0) | 3,841 (37.7) |  |
|  | Very good | 514 (14.2) | 1,096 (10.8) |  |
| IRH | |  |  | <0.001 |
|  | Moderately or severely ill | 22 (0.6) | 149 (1.5) |  |
|  | Slightly ill | 223 (6.2) | 1,484 (14.6) |  |
|  | Relative healthy | 2,142 (59.3) | 6,427 (63.1) |  |
|  | Healthy | 1,223 (33.9) | 2,130 (20.9) |  |
| CMWI | | -1.5 ([-3.2] - [-0.2]) | -1.5 ([-3.2] - [-0.2]) | 0.953 |

Notes: Values are presented as number (N) with percent (%) or medians with interquartile ranges (IQRs). BMI, body mass index. SRH, self-rated health. IRH, interviewer-rated health. CMWI, Chinese multimorbidity-weighted index.

# Table S4. Characteristics of participants included in analyses of changes of indicators with 4-year mortality from 2014 to 2018

| **Baseline Characteristics** | | **Died (*N*=928)** | **Survived or lost to follow-up (*N*=2,682)** | ***P*-value** |
| --- | --- | --- | --- | --- |
| Age, year | | 84.0 (77.0-90.0) | 74.0 (69.0-81.0) | <0.001 |
| Sex | |  |  | 0.010 |
|  | Male | 491 (52.9) | 1,285 (47.9) |  |
|  | Female | 437 (47.1) | 1,397 (52.1) |  |
| Residence | | |  | 0.969 |
|  | Rural | 579 (62.4) | 1,676 (62.5) |  |
|  | Urban | 349 (37.6) | 1,006 (37.5) |  |
| Education, years | | |  | <0.001 |
|  | Illiteracy | 526 (56.7) | 1,247 (46.5) |  |
|  | Primary education | 316 (34.1) | 1,000 (37.3) |  |
|  | Middle school or above | 86 (9.3) | 435 (16.2) |  |
| Annual income, yuan | | | | 0.850 |
|  | 0-29,999 | 718 (77.4) | 2,094 (78.1) |  |
|  | 30,000-59,999 | 121 (13.0) | 347 (12.9) |  |
|  | > 60,000 | 89 (9.6) | 241 (9.0) |  |
| Marital status | | | | <0.001 |
|  | Married or cohabitation | 557 (60.0) | 1,115 (41.6) |  |
|  | Unmarried or widowed | 371 (40.0) | 1,567 (58.4) |  |
| Physical activity | |  |  | 0.022 |
|  | No | 613 (66.1) | 1,658 (61.8) |  |
|  | Yes | 315 (33.9) | 1,024 (38.2) |  |
| Smoking history | | |  | 0.066 |
|  | Never smoking | 549 (59.2) | 1,679 (62.6) |  |
|  | Ever smoking | 379 (40.8) | 1,003 (37.4) |  |
| Drinking history | | |  | 0.189 |
|  | Never drinking | 582 (62.7) | 1,748 (65.2) |  |
|  | Ever drinking | 346 (37.3) | 934 (34.8) |  |
| BMI, kg/m^2^ | | 20.3 (18.3-22.4) | 21.2 (19.0-23.7) | <0.001 |
| SRH | |  |  | 0.013 |
|  | Poor or very poor | 146 (15.7) | 341 (12.7) |  |
|  | Fair | 300 (32.3) | 829 (30.9) |  |
|  | Good | 373 (40.2) | 1,107 (41.3) |  |
|  | Very good | 109 (11.8) | 405 (15.1) |  |
| IRH | |  |  | <0.001 |
|  | Moderately or severely ill | 7 (0.8) | 15 (0.6) |  |
|  | Slightly ill | 82 (8.8) | 141 (5.3) |  |
|  | Relative healthy | 564 (60.8) | 1,578 (58.8) |  |
|  | Healthy | 275 (29.6) | 948 (35.4) |  |
| CMWI | | -1.5 ([-3.2] - [-0.2]) | -1.5 ([-3.2] - [-0.2]) | 0.993 |

Notes: Values are presented as number (N) with percent (%) or medians with interquartile ranges (IQRs). BMI, body mass index. SRH, self-rated health. IRH, interviewer-rated health. CMWI, Chinese multimorbidity-weighted index.

# Table S5. Characteristics of participants included in analyses of trajectories of indicators with 4-year mortality from 2014 to 2018

| **Baseline Characteristics** | | **Died (*N*=645)** | **Survived or lost to follow-up (*N*=1,949)** | ***P*-value** |
| --- | --- | --- | --- | --- |
| Age, year | | 83.0 (76.0-89.0) | 74.0 (69.0-81.0) | <0.001 |
| Sex | |  |  | 0.004 |
|  | Male | 355 (55.0) | 943 (48.4) |  |
|  | Female | 290 (45.0) | 1,006 (51.6) |  |
| Residence | | |  | 0.079 |
|  | Rural | 404 (62.6) | 1,144 (58.7) |  |
|  | Urban | 241 (37.4) | 805 (41.3) |  |
| Education, years | | |  | <0.001 |
|  | Illiteracy | 353 (54.7) | 894 (45.9) |  |
|  | Primary education | 221 (34.3) | 721 (37.0) |  |
|  | Middle school or above | 71 (11.0) | 334 (17.1) |  |
| Annual income, yuan | | | | 0.756 |
|  | 0-29,999 | 502 (77.8) | 1,492 (76.6) |  |
|  | 30,000-59,999 | 85 (13.2) | 279 (14.3) |  |
|  | > 60,000 | 58 (9.0) | 178 (9.1) |  |
| Marital status | | | | <0.001 |
|  | Married or cohabitation | 372 (57.7) | 793 (40.7) |  |
|  | Unmarried or widowed | 273 (42.3) | 1,156 (59.3) |  |
| Physical activity | |  |  | 0.001 |
|  | No | 427 (66.2) | 1,148 (58.9) |  |
|  | Yes | 218 (33.8) | 801 (41.1) |  |
| Smoking history | | |  | 0.115 |
|  | Never smoking | 370 (57.4) | 1,187 (60.9) |  |
|  | Ever smoking | 275 (42.6) | 762 (39.1) |  |
| Drinking history | | |  | 0.603 |
|  | Never drinking | 406 (63.0) | 1,250 (64.1) |  |
|  | Ever drinking | 239 (37.1) | 699 (35.9) |  |
| BMI, kg/m^2^ | | 20.4 (18.3-22.6) | 21.4 (19.1-23.9) | <0.001 |
| SRH | |  |  | 0.020 |
|  | Poor or very poor | 103 (16.0) | 237 (12.2) |  |
|  | Fair | 208 (32.3) | 597 (30.6) |  |
|  | Good | 253 (39.2) | 806 (41.4) |  |
|  | Very good | 81 (12.6) | 309 (15.9) |  |
| IRH | |  |  | <0.001 |
|  | Moderately or severely ill | 7 (1.1) | 10 (0.5) |  |
|  | Slightly ill | 56 (8.7) | 101 (5.2) |  |
|  | Relative healthy | 386 (59.8) | 1,126 (57.8) |  |
|  | Healthy | 196 (30.4) | 712 (36.5) |  |
| CMWI | | -1.5 ([-3.2] - [-0.2]) | -1.5 ([-3.2] - [-0.2]) | 0.689 |

Notes: Values are presented as number (N) with percent (%) or medians with interquartile ranges (IQRs). BMI, body mass index. SRH, self-rated health. IRH, interviewer-rated health. CMWI, Chinese multimorbidity-weighted index.

# Table S6. Trajectory groups

|  | | **Trajectory Groups** | | | |
| --- | --- | --- | --- | --- | --- |
|  |  | **2** | **3** | **4** | **5** |
| ***SRH*** | | | | | |
|  | AIC | -11345.1 | -11286.7 | -11163.4 | -11072.1 |
|  | BIC | -11368.5 | -11321.9 | -11210.3 | -11130.7 |
| ***IRH*** | |  |  |  |  |
|  | AIC | -8887.4 | -8661.7 | -8368.9 | -8271.8 |
|  | BIC | -8910.8 | -8626.6 | -8415.8 | -8330.5 |
| ***CMWI*** | |  |  |  |  |
|  | AIC | -16987.7 | -16991.7 | -16556.2 | -16343.5 |
|  | BIC | -17011.1 | -17026.8 | -16603.0 | -16402.1 |

Notes: Trajectories were divided into 2, 3, 4, and 5, respectively, to compare their fit levels.


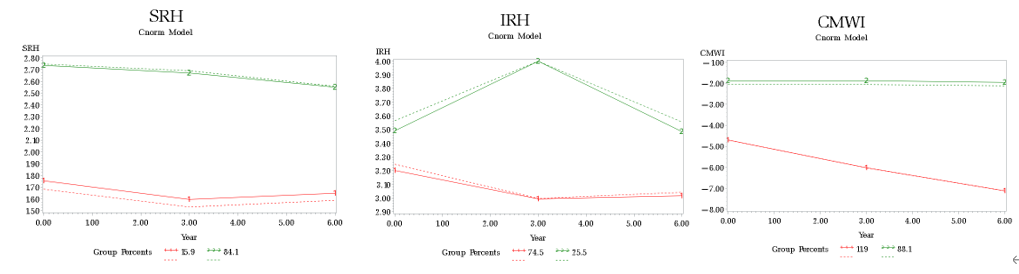


# Figure S1. Trajectories of SRH, IRH, and CMWI

Notes: SRH, self-rated health. IRH, interviewer-rated health. CMWI, Chinese multimorbidity-weighted index. “1” referred to "high SRH/IRH/CMWI". “2” referred to "low and declining SRH/IRH/CMWI".
